# Longchai Decoction Treated the Fibrosis of Liver Induced by CCl_4_ Regulates Nrf2/GPX4 Pathway to Suppress Ferroptosis

**DOI:** 10.1111/jcmm.71151

**Published:** 2026-04-25

**Authors:** Hui Wang, Liang Wang, Fangshi Zhu, Siru Chen

**Affiliations:** ^1^ Affiliated Hospital of Integrated Traditional Chinese and Western Medicine Nanjing University of Chinese Medicine Nanjing China; ^2^ Department of Rehabilitation Medicine The Affiliated Huai'an Hospital of Xuzhou Medical University and The Second People's Hospital of Huai'an Huai'an China; ^3^ Huai'an TCM Hospital Affiliated to Nanjing University of Chinese Medicine Huai'an China; ^4^ Department of Traditional Chinese Medicine The Affiliated Huai'an Hospital of Xuzhou Medical University and The Second People's Hospital of Huai'an Huai'an China

**Keywords:** ferroptosis, GPX4, liver fibrosis, longchai decoction, Nrf2

## Abstract

The aim of the study is to evaluate the therapeutic efficacy of Longchai Decoction (LCD), an empirical traditional Chinese medicine formula for liver disorders, on liver fibrosis (LF) and to elucidate its molecular mechanisms. A mouse model of LF and an erastin‐induced ferroptosis model was established in LX‐2 cells, which were then treated with silybin and LCD. The therapeutic function of LCD on liver fibrosis was evaluated using biochemical assays, histopathological staining, transmission electron microscopy, transcriptome sequencing, and immunohistochemistry. The mechanisms by which LCD alleviated LF were investigated using western blotting and immunofluorescence. LCD treatment significantly decreased the serum concentrations of IL‐1β, IL‐6, and TNF‐α. It also effectively suppressed lipid peroxidation, diminished iron accumulation, and preserved mitochondrial integrity within hepatic tissues. Transcriptome sequencing analysis revealed that LCD significantly increased the levels of genes including *NRF2*, *GPX4,* and *HO‐1*, thereby modulating the Nrf2/GPX4 pathway both in vivo and in vitro, thereby inhibiting lipid peroxidation and ferroptosis. Our findings suggest that LCD plays a protective role against hepatocyte ferroptosis and mitigates CCl4‐induced liver damage and fibrosis through the activation of the Nrf2/GPX4 pathway.

## 1 Introduction

The liver performs a vital metabolic function in the human body [1], and exerts a significant function in the detoxification of harmful substances, steroid synthesis, and the secretion of bile [2]. liver fibrosis (LF) is a prevalent pathological condition and its progression can lead to irreversible cirrhosis and, potentially, the development of liver cancer [3]. Epidemiological data indicate that over two million individuals worldwide succumb to liver disease annually, with 4% of these fatalities linked to fibrosis‐related liver disease [4]. The reversal or mitigation of LF has the potential to substantially decrease the rate of cirrhosis and liver cancer [5]. However, no effective antifibrotic agents are currently available in clinical practice. Consequently, it is imperative to investigate treatment modalities or strategies for anti‐LF that are both effective and low in toxicity.

Research has demonstrated a close correlation between ferroptosis and the occurrence of LF [6]. Ferroptosis represents a distinct mode of cell death that does not involve apoptosis and is reliant on iron, marked by the peroxidation of lipids. This occurs because of the reduced activity of glutathione peroxidase 4 (GPX4) and subsequent lipid oxidation [7]. Hepatic stellate cells (HSCs) contain high levels of iron, which are essential for inducing cell death via the ferroptosis pathway [8]. Excessive accumulation of iron in the liver in conjunction with ferroptosis increases the susceptibility of mouse models to LF induced by CCl_4_ [9]. Notably, the administration of ferroptosis inhibitors has been shown to be effective in reversing this pathological process [10]. The inhibition of ferroptosis is, consequently, an important strategy for the treatment of LF.

The significance of TCM for treatment of liver diseases has been increasingly recognized [11]. Longchai Decoction (LCD) [12] is an empirical prescription derived from the famous TCM physician, Jin Shi. LCD comprises a variety of medicinal components that demonstrate considerable therapeutic effects in treating liver diseases in clinical settings. However, to date, there are no relevant reports on whether LCD has a therapeutic effect on LF or investigations into its mechanism of action.

The purpose of this research was to examine the therapeutic function of LCD in vivo for LF and to further elucidate the associated mechanisms involved.

## 2 Material and Methods

### 2.1 Preparation of LCD

All herbal ingredients used in the preparation of LCD were provided by The Second People's Hospital of Huai'an. LCD contains eight types of traditional Chinese medicines: *Solanum nigrum* (15 g), vinegar *Bupleurum* (10 g), *Oldenlandia diffusa* (30 g), vinegar *glossy privet* fruit (12 g), Fructus *Gardeniae praeparatus* (10 g), *Sedum sarmentosum* (40 g), *Glycyrrhiza* (10 g), and *Scutellaria baicalensis* (10 g). Quality control checks for LCD were performed based on the method used by Yizhi Zhou [13]. LCD contains several index components, such as quercetin, wogonoside, salidroside, isoliquiritigenin, isoliquiritin, isorhamnetin, liquiritin, liquiritigenin, saikosaponin A, saikosaponin D, wogonin, and ursolic acid [14].

The eight herbal components were prepared in water at a weight‐to‐volume ratio of 1:10 for a duration of 30 min, followed by a boiling process lasting an additional 30 min to obtain the filtrate. After this initial extraction, the remaining materials were subjected to a second boiling in water at the same 1:10 (w/v) ratio for thirty minutes. The resulting filtrates from both extractions were then combined, resulting in a final concentration of 2.65 g of crude medicinal extract per millilitre.

### 2.2 Chemicals and Reagents

Silybin capsules were purchased from Tianjin Tasly SANTS Pharmaceutical Co. LTD (H20040299) for use as a positive control. CCl_4_ was obtained from Tianjin Damao Chemical Reagent Factory (20,171,117, Tianjin, China). Aspartate aminotransferase (AST, C009‐3‐2) and alanine aminotransferase (ALT, C010‐3‐1) were purchased from the Jiancheng Institute (Nanjing, China). Mouse interleukin‐1β (IL‐1β, EK201B), IL‐6 (EK206), and TNF‐α (EK282) were bought from Lianke Bioengineering Institute (Shanghai, China). Hyaluronic acid (HA, BY‐EM222211), type IV collagen (IV‐C, BY‐EM220024), laminin (LN, BYHS504768), and procollagen type III (PC III, BYHS501012) were obtained from BYabscience. The iron kit was purchased from Arigo Biotechnology Company (ARG81386, Arigo, China). H&E staining kit (C0189S) and Masson's kit (C0189S) were obtained from Beyotime Biotechnology. Anti‐collagen II (68j1348) and anti‐GAPDH (AF7021) antibodies were obtained from Affinity Biosciences. Anti‐Nrf2 (ab313825), anti‐Fn1 (ab268020), and anti‐haeme oxygenase 1 (HO‐1, ab68477) were obtained from Abcam Technology (Abcam, USA). Anti‐SMAD2 antibody (#AF3449) was obtained from Affinity Biosciences. The mouse anti‐GPX4 (sc‐166,437) antibody was obtained from Santa Cruz Biotechnology.

### 2.3 Animal Experiment

Male BALB/c mice, categorized as specific pathogen‐free (SPF) and weighing between 18 to 20 g, were obtained from Ruige Biotechnology Corporation located in Guangzhou, China (SCXK 2023–0059). These mice were maintained under controlled environmental conditions as described. The experimental protocols adhered strictly to the ethical standards set forth by the Animal Ethics Committee of Guangzhou University of Chinese Medicine (Approval No. 20241024013). Before the commencement of the experiments, the mice were acclimatized for a duration of one week.

The mice were randomized into four groups: Ctrl group (*n* = 10), CCl_4_ group (*n* = 10), receiving CCl_4_ from week 0–6; LCD low‐dose group (LCD‐L, *n* = 10), receiving oral LCD (7.5 g/kg) from weeks 3–6 and CCl_4_ from week 0–6; and LCD high‐dose group (LCD‐H, *n* = 10), receiving oral LCD (15 g/kg) from weeks 3–6 and CCl_4_ from week 0–6. The Ctrl and CCl_4_ groups were administered the same volume of 0.1% CMC‐Na solution.

The body mass of the mice was assessed biweekly throughout the duration of the study. Finally, rats were anaesthetised by inhalation of an overdose of isoflurane. Blood was obtained from the abdominal veins, and liver samples were gotten. Some of the liver tissue was soaked in 4% paraformaldehyde, and the remaining tissues were kept at −80°C.

### 2.4 Preparation of LCD‐Containing Serum

Male SD mice, aged 6 weeks with body weight between 180 to 200 g, were acquired from Ruige Biotechnology Corporation located in Guangzhou, China (SCXK 2023–0059). The mice were kept in a controlled environment as described above. The experimental protocols outlined herein adhered to the standards set forth by the Animal Ethics Committee at Guangzhou University of Chinese Medicine (Approval No. 20250715653–1). All mice had unrestricted access to a standard diet as well as sterile water.

The SD rats were randomly divided into blank and LCD groups. The LCD group was administered 11 g·kg ^−1^·d^−1^ of LCD, and the blank group was fed with distilled water. After administration by seven consecutive gavages, the rats were anaesthetised by inhalation of an overdose of isoflurane 2 h after the last drug administration. Blood was drawn from the abdominal aorta, stored in anticoagulant tubes, and centrifuged at 4°C, 1581 × g for 15 min. Finally, plasma from rats of the same group was pooled, filtered, and stored at −80°C.

### 2.5 Cell Culture

The LX‐2 cells were obtained from Pricella Biotechnology. These cells were maintained and cultured in DMEM as described{Chen, 2025 #12355}{Feng, 2025 #12358}.

### 2.6 CCK8 Assay

When LX‐2 cells reached 50% confluence, they were planted in 96‐well plates and treated with different concentrations of LCD‐containing serum (1%, 2%, 4%, 8%, 16%, and 32%) and stimulated with or without erastin (20 μM) for 24 h. Following this, the cells were incubated with 10 μL of the CCK8 reagent for a duration of 3 h. The absorbance was subsequently tested at a wavelength of 450 nm.

### 2.7 Biochemical Analysis

Serum was centrifuged at 3500 rpm for 15 min, and serum levels of ALT and AST were tested according to the standardized operation of the kit reagents (Jiancheng Bioengineering Institute, Nanjing, China).

### 2.8 Serum Inflammatory Factors and Fibrosis Indexes Test

The levels of IL‐1β, IL‐6, TNF‐α, and IL‐10 (Lianke Bioengineering Institute, Shanghai, China), and fibrosis indexes such as HA, IV‐C, LN, and PC (Boyan, Nanjing, China) were tested using ELISA kits.

### 2.9 Histopathological Examination and Masson's Trichrome Staining

Liver specimens underwent immersion in a 4% paraformaldehyde solution for a duration of 48 h before the processes of dehydration, paraffin embedding, and subsequent sectioning into slices measuring 4 μm in thickness. Following the sectioning, the tissue samples were dewaxed, rehydrated, and subjected to staining utilizing an H&E kit obtained from Beyotime. In addition, the method for Masson's trichrome staining was performed as described previously [15]. Positively stained areas were quantified using ImageJ (National Institutes of Health, Bethesda, MD, USA), and liver sections were imaged under a light microscope (Nexcope, NE620).

### 2.10 Immunohistochemistry

The liver sections embedded in paraffin were subjected to deparaffinization utilizing xylene and subsequently rehydrated through an ethanol gradient. The immunohistochemistry was performed as described{Chen, 2025 #12342}. The next phase involved incubating the slides overnight at 4°C with primary antibodies, including collagen II, Fn1, and SMAD2. Goat anti‐rabbit IgG H&L (HRP) was used as the secondary antibody for 1 h at room temperature. The substrate solution used was DAB reagent, while haematoxylin was utilized for counterstaining the sections. For microscopic examination, three random visual fields from each tissue section were chosen, and the positive regions were quantified utilizing ImageJ software.

### 2.11 Immunofluorescence

Cellular samples were gathered and subsequently fixed using 4% paraformaldehyde for a duration of 15 min. The cells were subjected to permeabilization through treatment with 0.1% Triton X‐100 for 6 min, followed by blocking with 5% BSA for 30 min. A wash with PBS was performed for 3 min. The samples were subsequently incubated overnight at 4°C with an array of anti‐Nrf2 antibodies at a dilution of 1:200. Following this, the samples were treated with secondary antibodies conjugated to Alexa Fluor 488 for a period of 2 h at room temperature, protected from light. To enable visualization of the nuclei, the sections were counterstained using DAPI, and the resulting fluorescent signals were assessed using a ZEISS LSM 700 microscope (ZEISS, Oberkochen, Germany).

### 2.12 Antioxidant Enzyme Activity (4‐HNE、GSH、MDA、SOD) and Iron Assay

The activities of GSH, MDA, and SOD in the serum or cell supernatants were tested using a biochemical test kit. The level of 4‐HNE (Nanjing Jiancheng Bioengineering Institute, Nanjing, China) was detected using ELISA kits according to the kit protocols. Iron levels were analysed using an iron assay kit (Arigo, Shanghai, China).

### 2.13 Transmission Electron Microscopy Analysis

Liver tissue samples were preserved in a 2.5% glutaraldehyde solution at 4°C for a duration of 24 h, followed by graded ethanol dehydration and fixed in 1% OsO_4_ for 1 h. Following fixation, the tissues were embedded in epoxy resin. Thin sections of the embedded specimens were produced using an ultramicrotome and were stained with uranyl acetate and lead citrate. Finally, the prepared specimens were examined using an electron microscope.

### 2.14 RNA‐Seq Analysis

Total RNA was extracted from the liver tissues using a TRIzol kit (Accurate Biotechnology, China). The RNA samples were sequenced by Guangzhou Gene Denovo Biotechnology (Guangzhou, China). Data were analysed using an online data analysis platform (http://www.omicsmart.com).

### 2.15 Western Blot

The proteins were extracted from liver tissues and cells by RIPA with protease inhibitors. The protein concentrations of the samples were determined using a BCA kit (Biosharp, Anhui, China). Immunoblotting was performed as described previously [16]. Nrf2 (SAB, USA), HO‐1 (SAB, USA), GPX4 (Santa Cruz, USA), and GAPDH (Affinity, USA) were used as primary antibodies. The protein samples were visualized using an ECL kit (Biosharp, Anhui, China) and subsequently analysed by ImageJ.

### 2.16 Statistical Analysis

The results are presented as means with standard deviations (SD). To evaluate the differences across groups, univariate analysis of variance (ANOVA) alongside Tukey's post hoc test was used. A P‐value threshold of less than 0.05 was established to indicate statistical significance.

## 3 Results

### 3.1 LCD Could Ameliorate CCl_4_ ‐Induced Liver Injury in Mice

**FIGURE 1:**
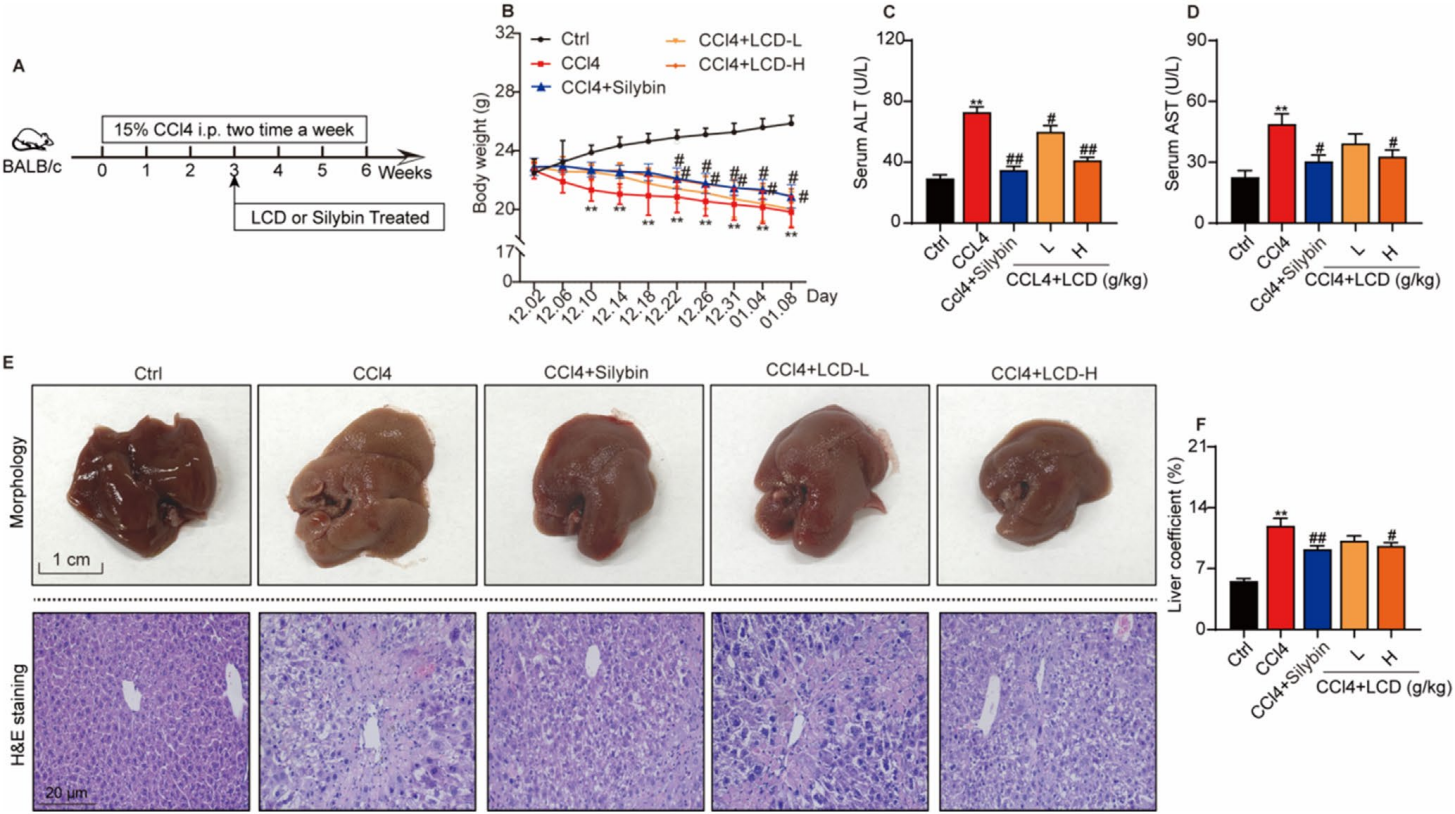
The effect of LCD on treating CCl_4_‐induced mice liver injury. (A) Experimental protocol flowchart. (B) Animal body weight (*n* = 9–10). (C) ALT levels in serum (*n* = 9–10). (D) Serum AST levels (*n* = 9–10). (E) Representative images of the liver and representative liver tissue pathology staining images (*n* = 9–10). (F) Liver coefficient (*n* = 8–10). Statistically significant differences were observed: *p < 0.05, **p < 0.01 in relation to the Ctrl. group, ^#^ *p* < 0.05 and ^##^ *p* < 0.01 compared to CCl4 group.

To evaluate the therapeutic effect of LCD in alleviating LF, we established a mouse model of LF by intraperitoneal injection of CCl_4_. Next, silybin and LCD were administered to the mice (Figure 1A). In Figure 1B, in comparison to the control group, a notable decrease in body weight was detected in mice treated with CCl4 beginning on day 9. However, both silybin and LCD treatment significantly inhibited body weight loss in the LF model. Furthermore, the levels of AST and ALT in serum were markedly increased in the model group, with obvious liver enlargement (Figure 1C,D), accompanied by conspicuous accumulation of nuclei around the hepatic sinusoids, sparse and irregularly arranged hepatocytes, extensive hepatocyte necrosis, and inflammatory cell infiltration (Figure 1E,F). Notably, following treatment with silybin and LCD in mice with LF, serum AST and ALT levels significantly decreased (Figure 1C,D), and liver weight was reduced. Histopathological analysis of liver tissues showed that silybin and LCD treatment significantly reduced inflammatory cell infiltration and maintained a more organized arrangement of hepatocytes and a more intact lobular structure in CCl_4_‐induced mice (Figure 1E,F). Collectively, the results revealed that LCD possessed a significant therapeutic function on CCl_4_‐induced liver injury in mice.

### 3.2 LCD Could Improve LF in CCl_4_ ‐Induced Mice

**FIGURE 2:**
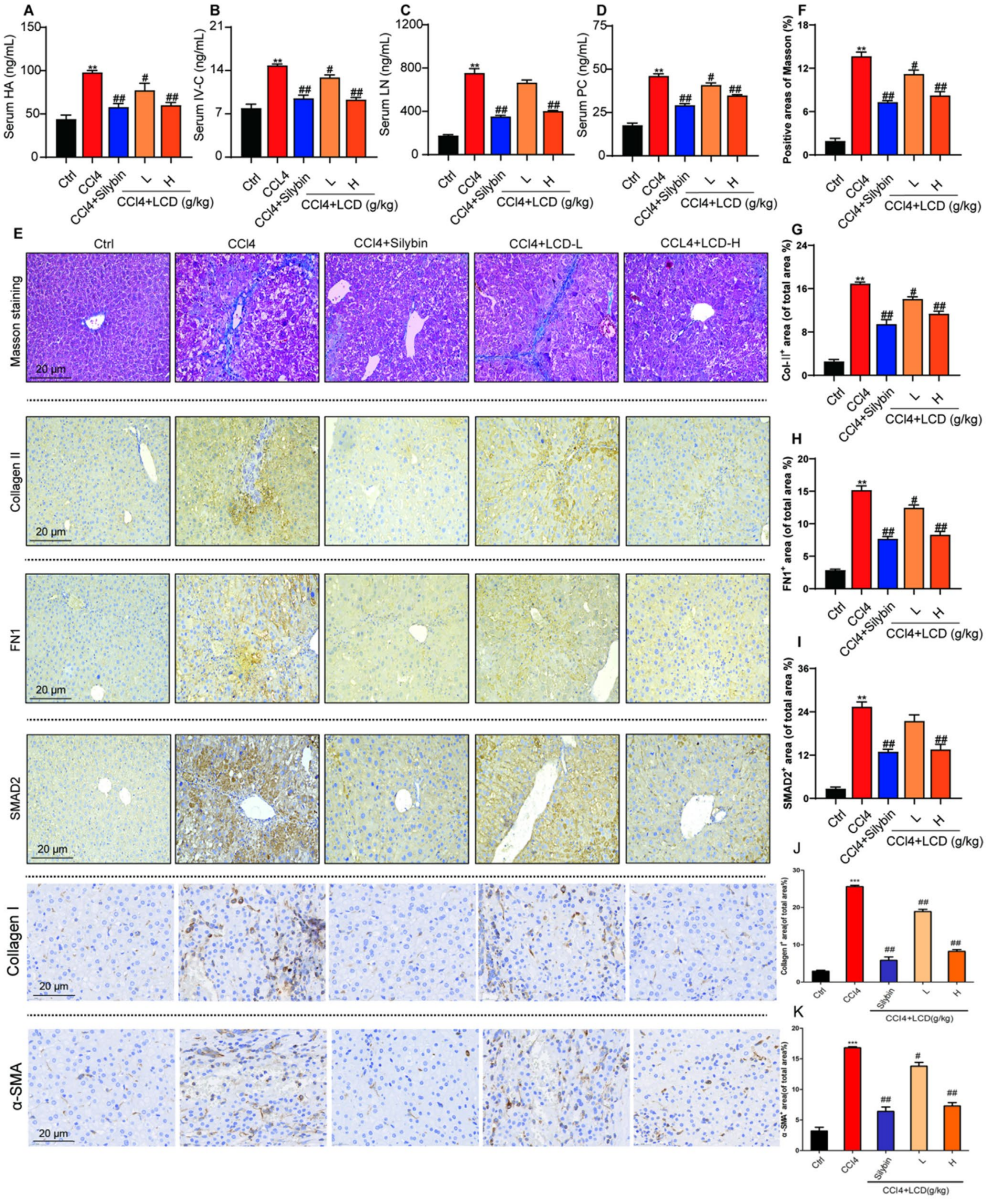
The effect of LCD on suppressing LF in CCl_4_‐induced mice. (A) Serum HA level. (B) Serum IV‐C level. (C) Serum LN level. (D) Serum PC III level. (E) Representative images of Masson staining, collagen II, Fn1, SMAD2, Collagen I, and α‐SMA proteins staining for liver tissues. (F) Masson staining positive area. (G) collagen II positive expression. (H) FN1 positive expression. (I) SMAD2 positive expression. (J) Collagen I positive expression. (K) α‐smooth positive expression. These results were shown as mean ± SD (*n* = 9–10). *p < 0.05, **p < 0.01 compared with the Ctrl group. ^#^ *p* < 0.05, ^##^ *p* < 0.01 compared with the CCl_4_ group.

To further elucidate the therapeutic effects of LCD on LF, we assessed the serum levels of key fibrosis biomarkers, namely, HA, IV‐C, LN, and PC III. As shown in Figure 2A–D, CCl_4_‐induced LF in mice resulted in a significant elevation in the serum levels of HA, IV‐C, LN, and PC III. Conversely, the administration of silybin and LCD caused a significant reduction in these serum biomarkers (Figure 2A–D). Furthermore, Masson's trichrome staining revealed that treatment with silybin and LCD in mice with LF markedly diminished the positively stained area in the liver (Figure 2E,F). Immunohistochemical analysis also showed that silybin and LCD markedly decreased the expression of collagen II, FN1, and SMAD2 (Figure 2E,G–I).

Next, we further performed IHC staining for the more classical fibrotic markers, type I collagen (Collagen I) and α‐smooth muscle actin (α‐SMA). Consistent with the above findings, LCD treatment significantly reduced the positive staining areas of both Collagen I and α‐SMA in the liver tissues compared to the CCl₄ model group (Figure 2J,K). Collectively, these results indicate that LCD exerts an effective therapeutic role on CCl₄‐induced LF by inhibiting hepatic stellate cell activation and extracellular matrix deposition.

### 3.3 LCD Inhibited Inflammation in the CCl_4_ ‐Induced Mice Model

**FIGURE 3:**
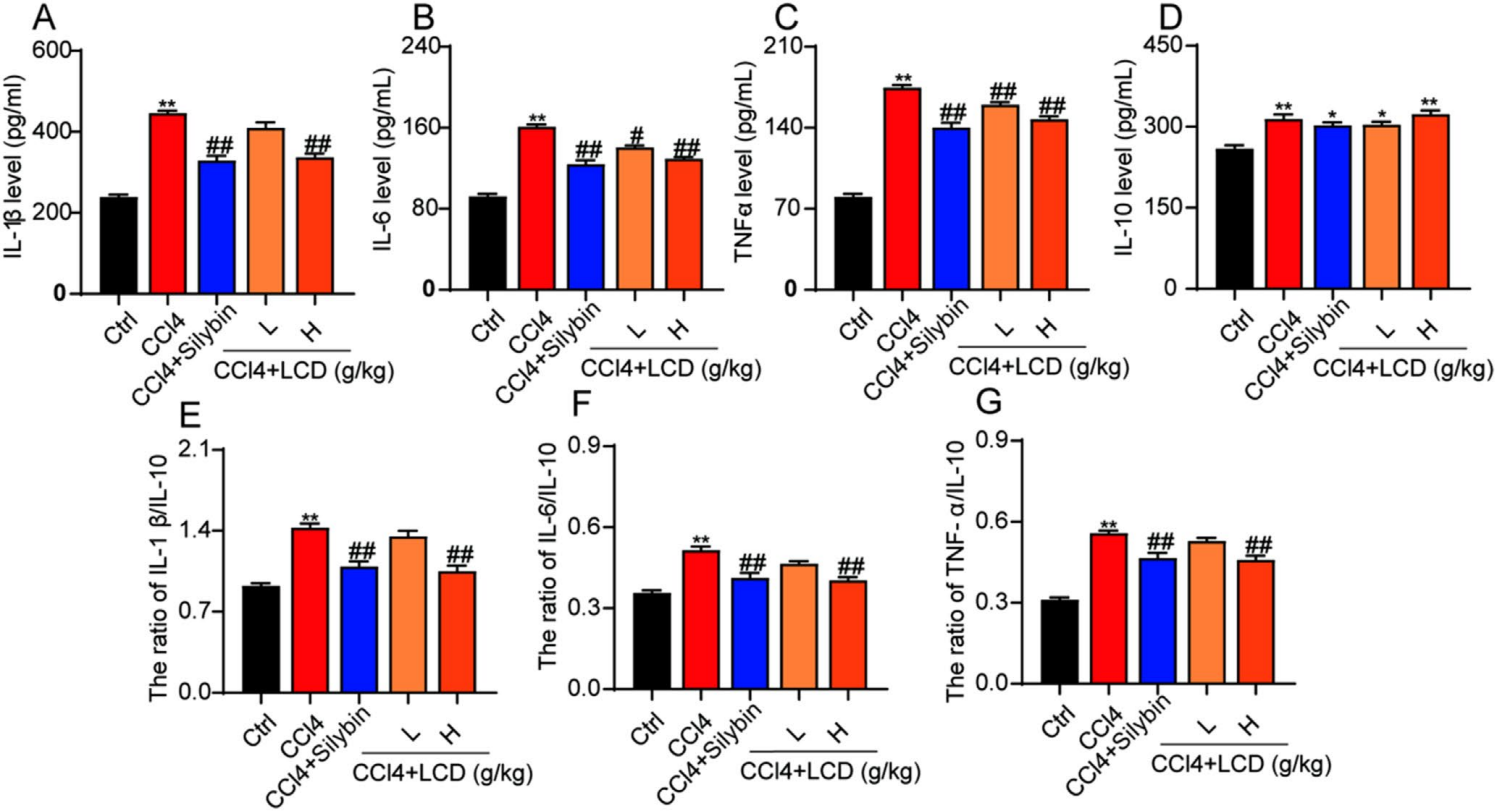
The effect of LCD on suppressing inflammation in CCl_4_‐induced mice. (A) The levels of serum IL‐1β were measured. (B) The concentrations of serum IL‐6 were evaluated. (C) The serum TNF‐α levels were determined. (D) Serum IL‐10 was analysed. (E) The IL‐1β/IL‐10 ratio was calculated. (F) The IL‐6/IL‐10 ratio was derived. (G) The TNF‐α/IL‐10 ratio was established.

Inflammation plays an important effect in the occurrence of LF [17]. We further analysed whether LCD mitigated CCl4‐induced LF in a mouse model by inhibiting inflammation. Compared to the control group, the serum levels of the cytokines, including IL‐1β, IL‐6, TNF‐α, and IL‐10, were significantly elevated in the mice with LF (Figure 3A–D). Following treatment with silybin and LCD, the serum levels of IL‐1β, IL‐6, and TNF‐α were significantly reduced, while the level of IL‐10 was significantly increased (Figure 3A–D). Notably, both silybin and LCD significantly decreased the ratios of IL‐1β/IL‐10, IL‐6/IL‐10, and TNF‐α/IL‐10 (Figure 3E–G), indicating that LCD exerts a significant inhibitory effect on inflammation in the CCl4‐induced LF mouse model.

### 3.4 LCD Could Suppress Lipid Peroxidation, Reduce Iron Levels, and Alleviate Mitochondrial Damage in CCl_4_ ‐Induced Mice

**FIGURE 4:**
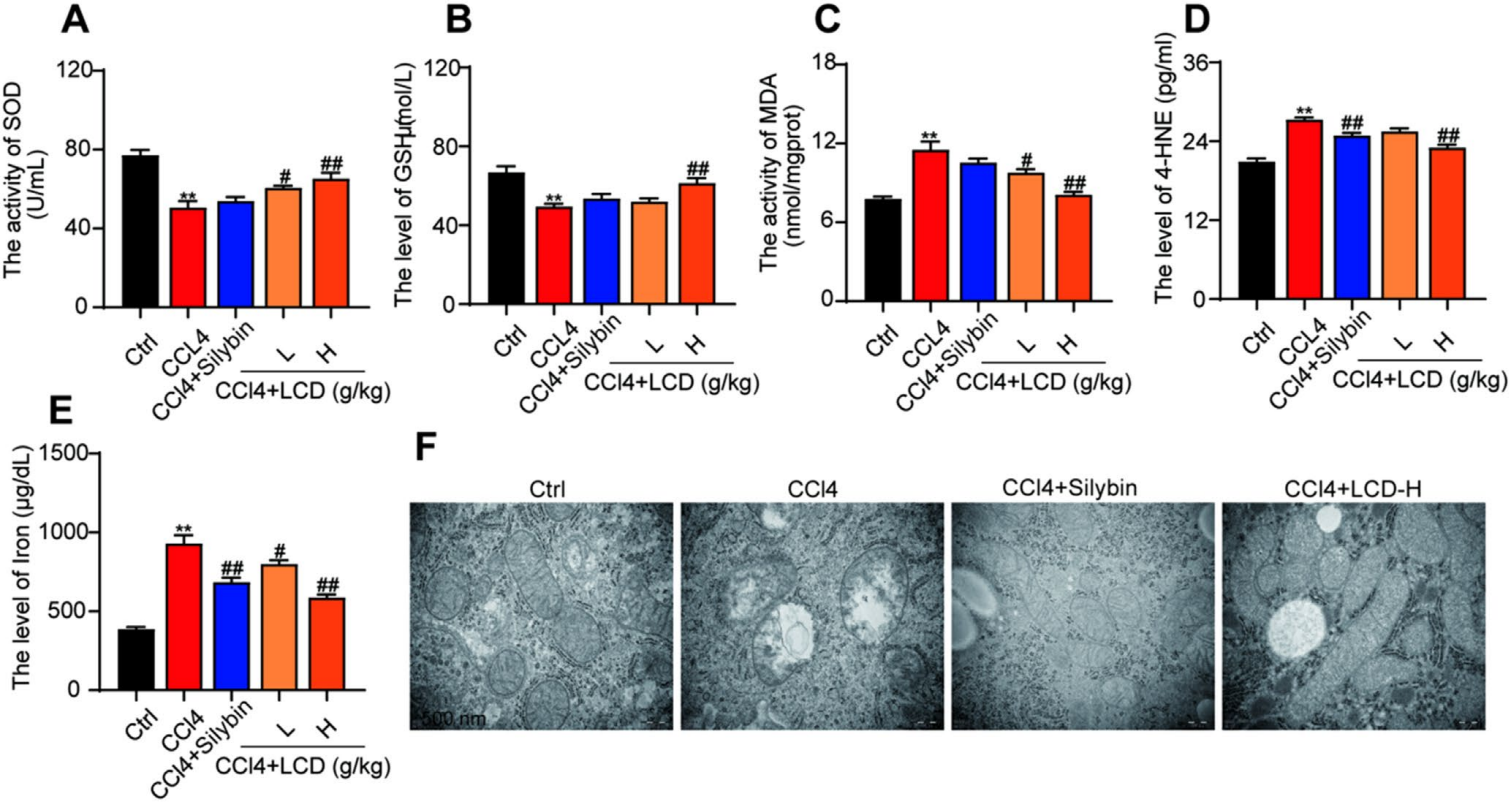
The effect of LCD on lipid peroxidation, Iron and mitochondria in CCl_4_‐induced mice. (A) The activity of SOD in serum. (B) The activity of GSH in serum. (C) The activity of MDA in serum. (D) The level of 4‐HNE in serum. (E) The level of Iron in serum. (F) Representative electron microscopic picture of the liver tissues (*n* = 3).

As shown in Figure 4A–D, the CCl_4_‐induced LF mouse model showed a significant decrease in the levels of SOD and GSH, while significantly increasing the levels of MDA and 4‐HNE. In contrast, treatment with LCD in the LF mouse model significantly increased the levels of SOD and GSH while inhibiting the levels of MDA and 4‐HNE (Figure 4A–D). Additionally, treatment with silybin significantly reduced the 4‐HNE levels but had no significant effect on the levels of SOD, GSH, and MDA (Figure 4A–D), suggesting that LCD inhibits lipid peroxidation in the LF mouse model. Furthermore, iron levels significantly increased in the LF mouse model (Figure 4E). Transmission electron microscopy results indicated that hepatocytes in this model exhibited reduced mitochondrial volume, increased membrane density, and diminished or absent mitochondrial cristae, along with damage to the outer mitochondrial membrane (Figure 4F). However, after treatment with LCD, there was a significant reduction in serum iron levels (Figure 4E), which preserved the number of liver mitochondria while maintaining the integrity of their structural morphology (Figure 4F).

### 3.5 Effect of LCD on Activating Nrf2/GPX4 Signalling Pathway and Suppressing Fibrotic Markers in CCl_4_ ‐Induced Mice

**FIGURE 5:**
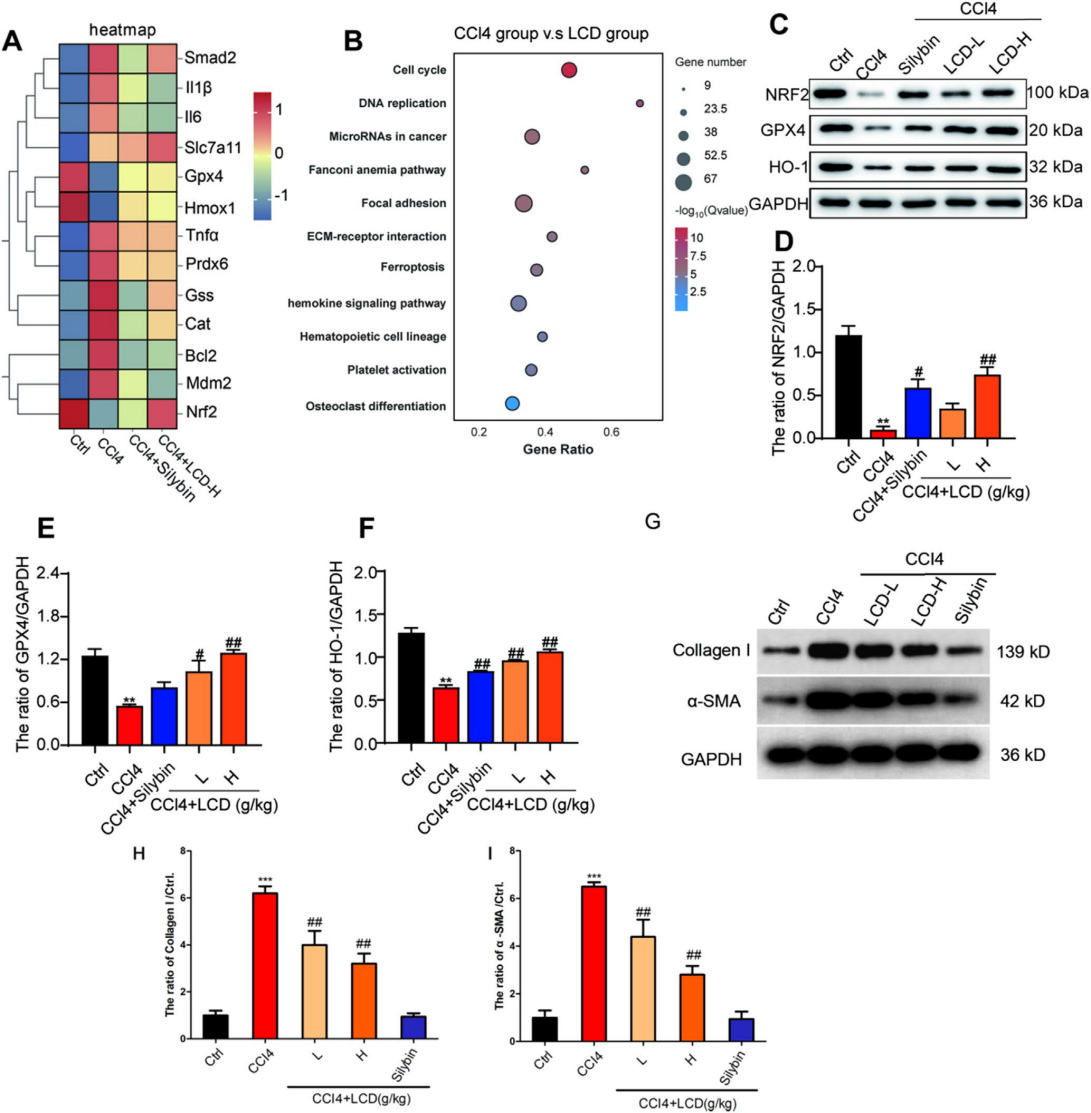
The impact of LCD on regulating Nrf2/GPX4 signalling pathway in CCl_4_‐induced mice. (A) Gene heat map in each group. (B) KEGG analysis between the CCl_4_ group and LCD group. (C) The representative immunoblot images for Nrf2, GPX4 and HO‐1 proteins in liver tissues. (D), (E), and (F) illustrate the expression levels of Nrf2, GPX4, and HO‐1 proteins, respectively, in the liver tissue. (G) The representative immunoblot images for Collagen I and α‐SMA proteins in liver tissues. (H) and (I) illustrate the expression levels of Collagen I and α‐SMA proteins in the liver tissue.

To further elucidate the mechanism of action of LCD in treating CCl_4_‐induced LF in mice, RNA sequencing was used to analyse the genome‐wide expression in the liver tissue. As shown in Figure 5A, the LF mouse model showed significantly upregulated expression of genes associated with fibrosis, apoptosis, and inflammation and downregulated expression of genes such as Nrf2 and GPX4. KEGG analysis indicated that LCD primarily influenced ferroptosis and oxidative stress pathways (Figure 5B). Consistently, western blotting results demonstrated that the LCD significantly upregulated the levels of Nrf2, GPX4, and HO‐1 proteins (Figure 5 C‐F). Furthermore, to directly verify the inhibitory effect of LCD on hepatic stellate cell activation and extracellular matrix deposition, we examined the protein levels of key fibrotic markers, type I collagen (Collagen I) and α‐smooth muscle actin (α‐SMA). The results showed that CCl₄ induction significantly upregulated the expression of Collagen I and α‐SMA in liver tissues, while LCD treatment (including both low and high doses) dose‐dependently and significantly downregulated the expression levels of these two proteins (Figure 5G–I). These findings indicate that LCD not only exerts antioxidant and anti‐ferroptosis effects by activating the Nrf2/GPX4 signalling pathway but also directly inhibits hepatic stellate cell activation (as evidenced by α‐SMA downregulation) and the excessive deposition of extracellular matrix (particularly type I collagen), thereby exerting anti‐liver fibrotic effects at multiple levels.

### 3.6 The Function of LCD on Cell Viability, Lipid Peroxidation, and Iron Accumulation in Erastin‐Induced LX‐2 Cells

**FIGURE 6:**
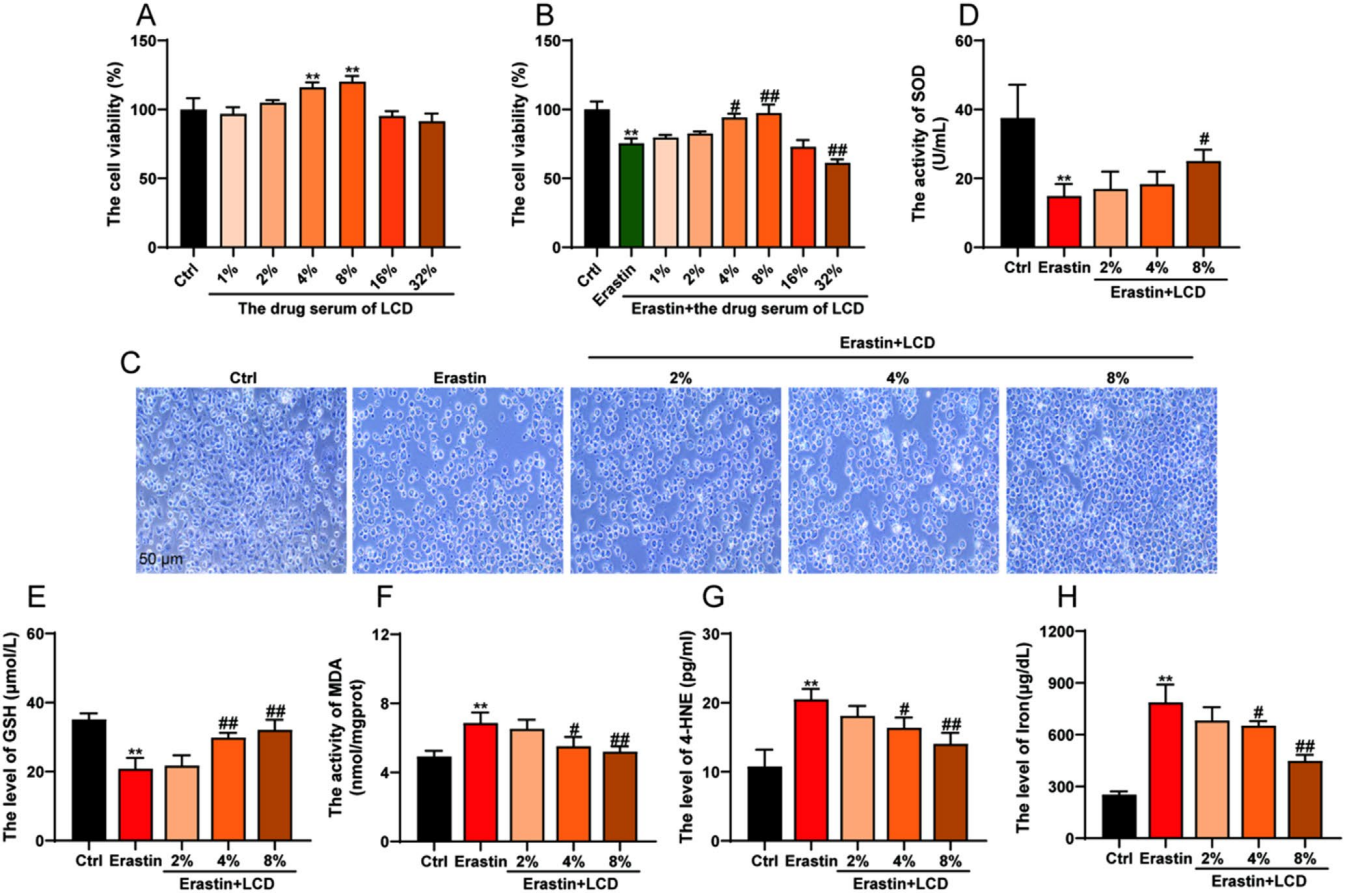
The role of LCD on regulating cell viability, lipid peroxidation and iron accumulation in LX‐2 cells. (A) The assessment of cell viability was conducted following treatment with varying concentrations of serum supplemented with LCD. (B) Cell viability was evaluated subsequent to the administration of erastin alongside different concentrations of serum containing LCD. (C) Representational images of LX‐2 cells subjected to treatment with 2%, 4%, and 8% of serum containing LCD are provided. (D) The measurement of superoxide dismutase (SOD) activity was performed. (E) The evaluation of glutathione (GSH) activity was carried out. (F) The concentration of malondialdehyde (MDA) was quantified. (G) The level of 4‐hydroxynonenal (4‐HNE) was assessed. (H) The concentration of iron was tested.

To further elucidate the mechanism of LCD in therapy of LF, an in vitro cell model was established using LX‐2 cells via erastin‐induced ferroptosis. The results of the cell viability assays showed that serum containing 1%–8% LCD enhanced the viability of LX‐2 cells in a dose‐dependent manner (Figure 6A). However, when the concentration of LCD‐containing serum exceeded 16%, cell viability was inhibited (Figure 6A). Additionally, erastin‐induced ferroptosis significantly reduced cell viability. Interestingly, serum containing 1%–8% LCD effectively counteracted erastin‐induced ferroptosis of the cells (Figure 6B). Compared to the erastin group, when the concentration of LCD‐containing serum exceeded 32%, the viability of LX‐2 cells was significantly inhibited (Figure 6B). Therefore, the drug concentrations of the LCD‐containing serum selected for intervention in LX‐2 cells were 2%, 4%, and 8%, as this range could mitigate the damage caused by erastin in LX‐2 cells in a dose‐dependent manner (Figure 6B,C). As shown in Figure 6D–G, the erastin‐induced ferroptosis in LX‐2 cells significantly decreased the levels of SOD and GSH, while increasing the levels of MDA and 4‐HNE (Figure 6D–G). After intervention with LCD‐containing serum in the erastin‐induced ferroptosis model in LX‐2 cells, there was a dose‐dependent increase in SOD and GSH levels, accompanied by a significant reduction in MDA and 4‐HNE levels (Figure 6D–G), suggesting that LCD can inhibit lipid peroxidation in the erastin‐induced ferroptosis model in LX‐2 cells. Furthermore, the erastin‐induced LX‐2 cell model showed significantly elevated levels of iron (Figure 6H), whereas LCD‐containing serum reduced these levels in a dose‐dependent manner (Figure 6H). Overall, these results indicated that LCD inhibited lipid peroxidation and reduced iron accumulation in the erastin‐induced ferroptosis model in LX‐2 cells.

### 3.7 LCD Could Activate Nrf2/GPX4 Pathway in Erastin‐Induced LX‐2 Cells

**FIGURE 7:**
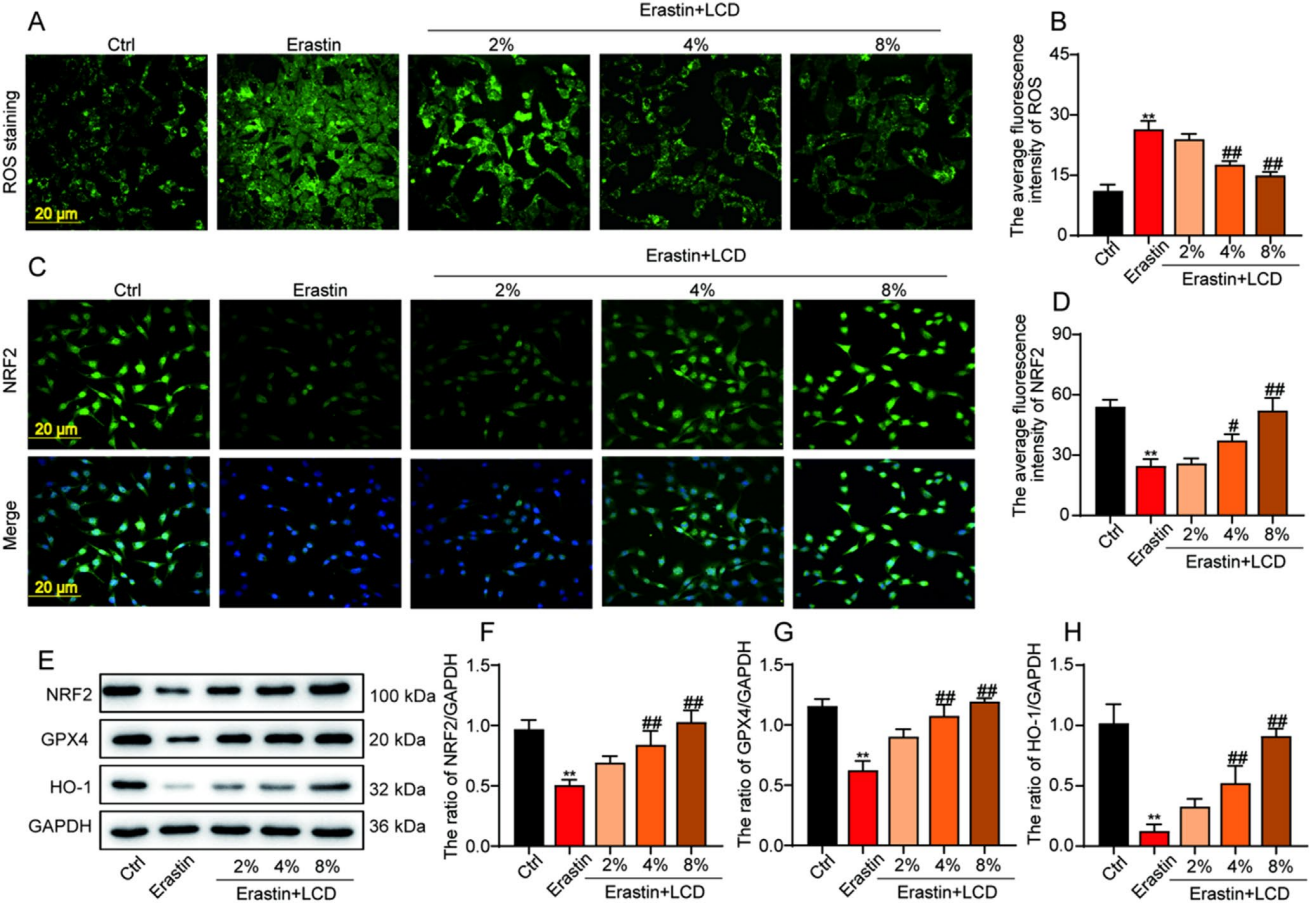
The effect of LCD on regulating Nrf2/GPX4's pathway in erastin‐induced LX‐2 cells. (A) The representative images for ROS in erastin‐induced LX‐2 cells. (B) The expression intensity of ROS fluorescence. (C) The representative images for Nrf2 protein in erastin‐induced LX‐2 cells. (D) The expression intensity of Nrf2 fluorescence. (E) The representative immunoblot images for Nrf2, GPX4, and HO‐1 proteins in erastin‐induced LX‐2 cells. (F) The expression of Nrf2 protein analysed by Image J software. (G) The expression of GPX4 protein analysed by Image J software. (H) The expression of HO‐1 protein analysed by Image J software.

Immunofluorescence staining was performed to assess the role of LCD on Nrf2 expression in an erastin‐induced LX‐2 cell model. The data revealed that the erastin‐induced LX‐2 cell model exhibited significantly reduced expression and nuclear translocation of Nrf2 protein (Figure 7A,B). However, following intervention with LCD‐containing serum, there was an increase in a dose‐dependent manner in both the expression and nuclear translocation of the Nrf2 protein (Figure 7C,D). Furthermore, western blot analysis revealed that LCD‐containing serum enhanced the level of Nrf2, GPX4, and HO‐1 relying on the dose (Figure 7E–H). Taken together, these data revealed that LCD activated the Nrf2/GPX4 pathway to suppress ferroptosis in liver cells.

## 4 Discussion

LF represents a significant phase in the advancement of chronic liver disorders, easily forming cirrhosis and hepatocellular carcinoma. Currently, there is a scarcity of effective therapeutic drugs available for clinical practice [18]. LCD, an empirical formula developed by Professor Jin Shi, is primarily used to treat liver diseases. However, there are no relevant reports on its therapeutic effects on LF. The study is the first confirmation of the role of LCD on CCl_4_‐induced LF and additionally investigates the mechanism by which LCD alleviates LF via Nrf2/GPX4 pathway to suppress hepatocyte ferroptosis.

The classic CCl_4_‐induced LF model is widely used to study the pathogenesis of LF and evaluate antifibrotic drugs [19]. CCl_4_ activates the production of free radicals through cytochrome P450 in liver cells, leading to lipid peroxidation and the generation of ROS, which cause liver cell death and contribute to LF [20]. Our data demonstrated that LCD significantly mitigated CCl_4_‐induced hepatotoxicity in mice by preventing weight loss, reducing serum levels of AST and ALT, improving liver morphology, and alleviating pathological damage to the liver tissue, including the suppression of liver inflammation, cytoplasmic vacuolation, and cellular necrosis. Importantly, LCD treatment significantly reduced the levels of HA, IV‐C, LN, and PC III in serum of the CCl_4_‐induced mouse model, decreased Masson staining in the liver tissue, and inhibited the expression of proteins such as collagen II, Fn1, and SMAD2 in the liver tissues.

It is reported that CCl_4_ treatment can induce liver inflammation, a significant factor that causes liver damage and promotes LF [21, 22]. In conjunction with the histopathological results from the liver, we further analysed the function of LCD on inflammation in vivo. The data revealed that treatment with LCD significantly decreased the secretion of inflammatory cytokines. This indicates that LCD can inhibit inflammation and ameliorate CCl_4_‐induced LF.

An increasing number of studies showed that ferroptosis in hepatocytes is closely related to the development of various liver diseases, including steatohepatitis, fibrosis, and hepatocellular carcinoma [23]. HSCs contain a substantial amount of iron that is essential for iron‐induced cell death. Excess iron generates Fe2+ in the body, which initiates the Fenton reaction, leading to the production of hydroxyl radicals (•OH) that directly attack lipids. This process causes the peroxidation of polyunsaturated fatty acids and subsequent iron‐induced cell death [24]. Nrf2 is a crucial transcription factor that regulates cellular oxidative stress and exerts a vital function in maintaining intracellular redox homeostasis. It can induce and modulate the composition and expression of antioxidant proteins, thereby reducing ROS damage to cells and preserving the body's redox balance, which contributes to protection against ferroptosis [25]. GPX4, a selenium‐containing enzyme, inhibits lipid peroxidation‐induced ferroptosis and is a significant antioxidant transcriptional target of Nrf2 [26]. An increasing number of studies have confirmed that the Nrf2‐GPX4 signalling pathway can ameliorate LF by inhibiting ferroptosis [27]. It has been reported that CCl_4_‐induced damage leads to excessive production of hydroxynonenal from the peroxidation of polyunsaturated fatty acids, as well as the oxidation and breakdown of cholesterol, which disrupts redox homeostasis and results in ferroptosis [28]. Here, we found that LCD significantly decreased lipid peroxidation and markedly upregulated the levels of proteins associated with the Nrf2‐GPX4 pathway in both a CCl_4_‐induced LF model and an erastin‐induced LX‐2 cell model, thereby exerting an inhibitory effect on hepatocyte ferroptosis.

## 5 Conclusion

The findings of this investigation indicated that LCD possesses the capability to suppress ferroptosis in hepatocytes and mitigated LF via activating the Nrf2/GPX4 pathway.

## Author Contributions

**Siru Chen:** writing – review and editing, project administration, methodology, conceptualization, investigation, funding acquisition, validation, supervision. **Fangshi Zhu:** writing – review and editing, conceptualization, validation, supervision, resources, software. **Hui Wang:** conceptualization, writing – original draft, validation, formal analysis, data curation, supervision, methodology, visualization. **Liang Wang:** software, formal analysis, supervision, investigation, validation.

## Funding

The authors have nothing to report.

## Disclosure

Generative AI statement: The authors declare that no Generative AI was used in the creation of this manuscript.

## Ethics Statement

The experimental protocols adhered strictly to the ethical standards set forth by the Animal Ethics Committee of Guangzhou University of Chinese Medicine (Approval No. 20241024013).

## Conflicts of Interest

The authors declare no conflicts of interest.

## Data Availability

The data that support the findings of this study are available from the corresponding author upon reasonable request.
